# Comparative Study of Three-Dimensional Stress and Crack Imaging in Concrete by Application of Inverse Algorithms to Coda Wave Measurements

**DOI:** 10.3390/s20174899

**Published:** 2020-08-30

**Authors:** Hanwan Jiang, Hanyu Zhan, Ziwei Ma, Ruinian Jiang

**Affiliations:** 1 Department of Civil Engineering, University of Wisconsin-Platteville, Platteville, WI 53818, USA; jianghan@uwplatt.edu; 2 Klipsch School of Electrical and Computer Engineering, New Mexico State University, Las Cruces, NM 88003, USA; 3 Department of Mathematics, University of Tennessee at Chattanooga, Chattanooga, TN 37403, USA; ziwei-ma@utc.edu; 4 Department of Engineering Technology & Surveying Engineering, New Mexico State University, Las Cruces, NM 88003, USA; rjiang@nmsu.edu

**Keywords:** coda wave, transducer network, large concrete beam, NDT, sensitivity kernel, inverse algorithm, three-dimensional crack and stress imaging

## Abstract

The intrinsic heterogeneity property of concrete causes strong multiple scatterings during wave propagation, forming coda wave that follows very complex trajectories. As a superposition of multiply scattered waves, coda wave shows great sensitivity to subtle changes, but meanwhile lose spatial resolution. To make use of its sensitivity and turn the limitation into advantage, this paper presents an experimental study of three-dimensionally imaging local changes in concrete by application of inverse algorithms to coda wave measurements. Load tests are performed on a large reinforced concrete beam that contains multiple pre-existing millimeter-scale cracks in order to match real life situation. The joint effects of cracks and stresses on coda waves have been monitored using a network of fixed transducers placed at the surface. The global waveform decorrelations and velocity variations are firstly quantified through coda wave interferometry technique. Subsequently, two inverse algorithms are independently applied to map the densities of changes at each localized position. Using this methodology, the stress changes and subtle cracks in the concrete beam are detected and imaged for both temporal and spatial domains.

## 1. Introduction

Concrete structures such as roads, dams, buildings, and bridges play extraordinary roles in this world. Mechanical stress is one of the most frequently reasons for initiating concrete cracking or micro-cracking. Much of the damage at early stage begins with scales that are smaller than the size of the majority of the aggregate. However, if they are continuously subject to overloading as well as chemical and thermal effects, they may extend rapidly in length, width, and number, and further lead to severe damages, even to structural failure. Developing nondestructive evaluation and testing (NDT) approaches to detect subtle damages and stresses within a structure, especially for a large number of aging infrastructures, are in great demand.

In literature, multitudes of techniques have been developed for concrete structural health monitoring (SHM) and NDT [1], through the use of ultrasound [2,3], ground penetration radar [4], sensor [5], laser [6,7], and optical fiber [8,9]. Among the various methods, ultrasonic wave has been used for decades to monitor concrete elements by applying the direct relationship between its velocity and concrete quality, mostly on a laboratory scale. That is, the porosity and cracking tend to reduce wave velocity, and therefore higher velocity usually reflects better condition of concrete [2]. Their main applications include the assessment of thickness, delamination, strength, and free-thaw resistance by measuring velocity, amplitude, and nonlinearity of direct wave [2]. However, the applicability of these techniques is limited to short transit time/distance between sensors and low ultrasound frequency (i.e., low sensitivity to micro-defects) due to intrinsic scattering property of concrete. The multiple scatterings significantly attenuate the energy of direct wave in a process similar to heat diffusion. The energy is not lost, but transformed into long-lasting waveforms following very complex trajectories, forming the so-called coda wave that evolve in space and time according to the diffusion equation [10]. Because the travel paths and transit time of coda wave are much longer and they may traverse the regions of interest repeatedly, the effects of minute changes occurring in a relative large volume of concrete accumulate to notable alteration in their waveforms. To make the best of the great sensitivity, coda wave interferometry (CWI) quantifies the alteration by computing its decorrelation (*DC*) and velocity variation (*dv*/*v*). Further analysis of the *DC* and *dv*/*v* values and dependence can distinguish different types of changes (i.e., damage and stress) and indicate the extent of changes [11]. But as a superposition of multiply scattered waves from different trajectories, coda wave has its own limitations of losing directional and spatial information about changes, thus locating changes in concrete remains a big challenge. The coda wave-based techniques can generally be separated into two categories depending on practical applications: evaluation of global structural health [12,13,14,15,16,17] and characterization of damages [18,19,20,21,22]. The first category focuses on global structure monitoring through the use of CWI [11] or diffusion equation [10] to quantify coda signal changes. Then with the help of sensor network [17] and/or further analysis, such as Mahalanobis distance [15] and diffusivity [16], the location, type, and magnitude of changes can be estimated up to a certain extent. The other category aims at identifying the locations, depths, and numbers of damages with a high degree of accuracy. Its general concept is also first to calculate coda waveform variations. An inversion procedure is then adopted to calculate the density of changes at each localized position through inverse algorithms, such as maximum likelihood [18], sensitivity kernel [19,20,22,23] and interpolation [21], resulting in maps of density of changes in stress and micro-structures. For additional details on the techniques, we refer the reader to the literature reviews published in refs. [3,11].

In this paper, we present an experimental study of three-dimensionally imaging local changes and damages in a large reinforced concrete beam that contains multiple pre-existing subtle cracks. A four-point bending test was conducted on the beam to match real life situation; i.e., during the experiment, the beam experiences the joint effects of subtle cracks and external loads. Coda waves were measured with a network of ultrasonic transducers placed at the surfaces, and further analyzed by coda waveform interferometry. Subsequently, following the previous inversion studies [19,20,22,23], two iterative algorithms are independently applied to the global parameters of *DC* and *dv*/*v* to map the density of changes. The results show that both *DC*- and *dv*/*v*-inversed images are capable of detecting stress changes and subtle cracks.

## 2. Materials and Equipment

### 2.1. Concrete Beam

A concrete beam with the volume V = 596 × 87 × 30 cm^3^ was made of Portland Type I/II cement, water, sand, aggregates, grade II fly ash, the furnace blast slag, and steel bar. After casting, it was maintained in a curing room of 20 ± 2 °C and 95% humidity following GB/T50081-2002 standard for four weeks. The ultimate compressive strength of the concrete was about 42.5 MPa by measuring 15 × 15 × 15 cm^3^ cubes made of the same composites and mixtures [14]. There were seven longitudinal hot rolled ribbed steel rebar with 25 mm diameter embedded at the bottom, and the design tensile strength of each rebar was 280 MPa.

It is worth noting this beam, as a duplicate of real beams utilized on a short-span bridge, itself could not hold the standard vehicle load. However, with the cast-in-place reinforced concrete deck and properly designed hinges that binding multiple adjacent beams to resist the load jointly, the bridge owns sufficient capacity to meet the criterion of the service deflection. The beam is selected as our first test subject to validate the feasibility of the approaches in well-controlled laboratory conditions.

### 2.2. Transducer Setup

**Figure 1:**
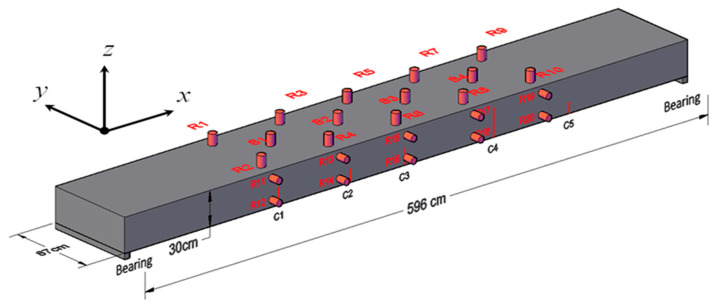
Ultrasonic transducer setup. S1~S4 label the source positions and R1~R20 label the receiver positions.

The ultrasonic transducer RS-2A designed by Softland Times Scientific & Technology Co. Ltd. has broadband working frequency of 60~400 kHz. It is 15 mm in thickness and 18.8 mm in diameter. A total of 24 RS-2A transducers were glued on the beam using the coupling medium of high vacuum silicone grease, as shown in Figure 1. Four transducers worked, as sources S1~S4 were positioned along the center line of the top surface, and the rest 20 transducers served as receivers R1~R20 were positioned in a 4 × 5 grid. In particular, the receivers were mounted along four parallel lines approximately at sections of *x* = L/4, 3L/8, L/2, 5L/8, and 3L/4. At the side surface, for each section there were two receivers placed at *z* = H/6 and 5H/6. At the top surface, for each section two receivers were placed at *y* = W/9 and 8W/9. Here, L = 596 cm, H = 30 cm, and W = 87 cm are the length, height, and width of the beam, respectively.

**Figure 2:**
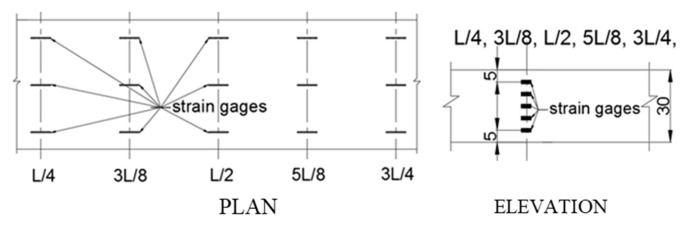
The plan and elevations views of the strain gauge setups.

Forty strain gauges were also installed on the top and side surfaces at sections of *x* = L/4, 3L/8, L/2, 5L/8 and 3L/4, see Figure 2. For each section, there were five gauges positioned at *z* = H/6, 2H/6, 3H/6, 4H/6, and 5H/6 of the side surface, and three gages were positioned at *y* = W/9, W/2, and 8W/9 of the top surface. The gauge measurements are accomplished with a DH3821 multi-channel static strain test and analysis system, and they are used to monitor and analyze the stress conditions of the beam during the experiment.

### 2.3. Excitation and Measurement Equipment

The excitation signal was generated by a high-power ultrasonic system RITEC SNAP 5000. The response signals that were detected by each receiver were amplified by an independent Smart AE amplifier, and then collected with a customer-made DS6-32B Multi-channel Holographic Acoustic Signal Analyzer. Each source-receiver pair has time trigger and counter to record the excitation and observation time.

## 3. Acoustical Measurements

After transducer and equipment setup, in the preloading step a force beyond the serviceability was conducted on the beam to create casual cracks, and the force was then released. Figure 1 illustrates the positions of five cracks C1~C5 appearing at one side surface, and their sizes under 0 kN were visually inspected and are listed in Table 1. These cracks with the widths on the order of 0.1 mm can be treated as typically pre-existing, subtle defects in concrete structures.

**Figure 3:**
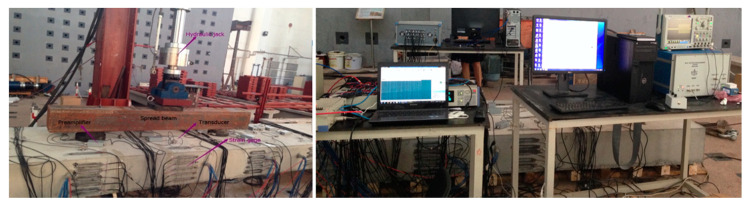
Photographs of (**left**) experimental setup and (**right**) excitation and measurement equipment.

Four-point bending tests were performed step-by-step on the beam, as shown in Figure 3. The loading levels, controlled through a hydraulic jack positioned in the middle of a 130 cm spread beam, was increased from 0 kN to 130 kN with a fixed increment of 10 kN. At each loading step *k*, successive excitations-acquisitions of wave signals were made for all available source-receiver pairs. Specifically, each loading step lasted 5 min, where the first 1 min. was utilized to stabilize the stress conditions in concrete before data collections. Subsequently, for each measurement, only one source was activated to generate the identical ultrasonic impulse at 150 kHz every *T* = 1 s, and all the receivers *R_j_* continuously recorded the response signals at 1.25 MHz sampling frequency. The excitation was reproduced 60 times in order to increase signal-to-noise ratios. The waveforms used for data processing was the average of 60 signals *h_i,j_^k^*(*t*) observed consecutively within one minute, and their standard deviations were utilized to estimate the noise levels of measurements. Then this source was turned off and another source was switched on to repeat the above procedure until all source-receiver pair measurements (lasting four minutes) were completed. In a word, each loading step includes 80 measurements corresponding to 4 × 20 source-receiver pairs and each measurement contained 60 full coda waveforms. Because the signals were observed in an extremely short time in a climate-controlled laboratory, the environmental conditions can be treated as constants. Consequently, the waveforms within each measurement cycle have an extremely high degree of similarity, and their correlation values generally are greater than 0.998.

## 4. Methodology and Data Processing

### 4.1. Coda Wave Interferometry

Coda wave has traveled a large volume for a long time, so that any small changes in concrete result in a notable waveform change. Coda wave interferometry (CWI), a technique initially developed from seismology, is able to quantify this waveform change through two parameters of decorrelation *DC* and relative velocity variation *dv*/*v* [11]. In this study, coda signals collected during whole propagation period *T* are directly used for comparison without time-window selection. This operation simplifies the data processing, but slightly reduces the CWI sensitivity since the changes at the earlier parts of coda waveforms are relatively small. The way to overcome this issue is to calculate stepwise changes by using just the previous measurement (same source-receiver pair but previous load) as reference. Then the stepwise velocity variations are multiplied to obtain the cumulative changes relative to the initial state (0 kN applied force) of experiment [12]. The stepwise CWI is expressed as

(1) $$DC_{i,j}^{k}=1-\frac{\int_{0}^{T}h_{i,j}^{k}\left\{t\left[1-{\left(dv/v\right)}_{i,j}^{k}\right]\right\}h_{i,j}^{k-1}\left(t\right)dt}{\sqrt{\int_{0}^{T}{\left(h_{i,j}^{k}\left\{t\left[1-{\left(dv/v\right)}_{i,j}^{k}\right]\right\}\right)}^{2}dt\int_{0}^{T}{\left[h_{i,j}^{k-1}\left(t\right)\right]}^{2}dt}}$$

The value of *dv*/*v* is determined by minimizing the *DC* value that makes two waveforms best resemble. Different types of changes can be distinguished through the interpretation of *DC* and *dv*/*v* dependence [11]. If there are changes as stress existing, by selecting appropriate *dv*/*v* values the phase shifts between waveforms can be removed; consequently, *DC* remains small values. However, cracks or defects that worked as extra scatters will induce waveform distortions, and thus *DC* keeps at large values irrespective of *dv*/*v*.

**Figure 4:**
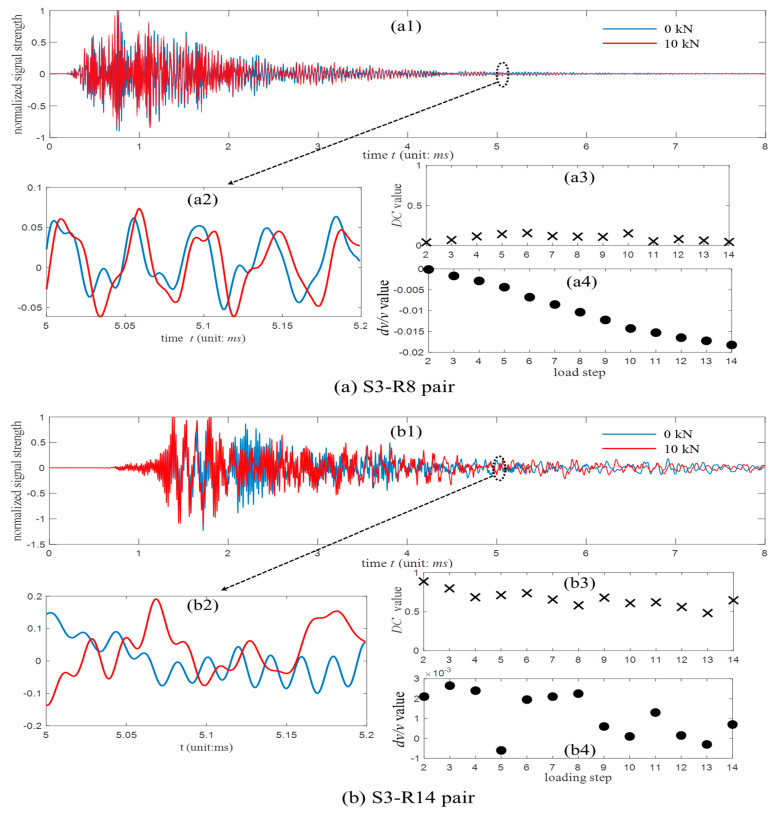
The waveforms as well as the decorrelation (*DC*) and *dv*/*v* values collected with the (**a**) S3-R8 and (**b**) S3-R14 pairs under varied loads.

Figure 4 shows an example of the coda signals that were collected with the S3-R8 and S3-R14 pairs. Because the S3-R8 pair is positioned far from the pre-existing cracks, the waveform in red shows an obvious stretching behavior as compared to the reference data in blue. Their calculated *DC* values are small under all the loads, and the *ε* values decrease almost linearly as the loads increase, see Figure 4(a3,a4). In contrast, the receiver R14 is placed close to the crack C2. The obvious waveform distortions are found in Figure 4(b1,b2). Their *DC* values keep large and *ε* varies between positive and negative values.

### 4.2. The Direct Problem

As coda wave is a superposition of partial waves over all possible trajectories, *DC* and *dv*/*v* values are the summations of defect in each local micro-structure *σ*(*r*) and velocity variation *m*(*r*) at each position *r* = (*x*, *y*, *z*), respectively. In other words, great sensitivity of coda wave to large volumes of media is accompanied with the reduction of spatial resolution about changes. Specifying detailed propagation trajectories is almost impossible. Fortunately, diffuse sensitivity kernel *K_i,j_*(*r*, *t*), derived based on energy transport can statistically describe the three-dimensional time-of-flight distributions of coda waves emitted by source *S_i_* at position *r_i_*, passing location *r,* and measured by receiver *R_j_* at position *r_j_* [14]. Thanks to this model, the global parameters of *DC* and *dv*/*v* are related to local property changes *σ*(*r*) and *m*(*r*) by

(2a) $$DC_{i,j}^{k}=\int_{0}^{V}\sigma^{k}\left(r\right)\cdot \frac{c^{k}}{2}\cdot K_{i,j}^{k}=\int_{0}^{V}\sigma^{k}\left(r\right)\cdot {\left(G_{DC}\right)}_{i,j}^{k}$$

(2b) $${\left(dv/v\right)}_{i,j}^{k}=\int_{0}^{V}m^{k}\left(r\right)\cdot \frac{K_{i,j}^{k}}{t}=\int_{0}^{V}m^{k}\left(r\right)\cdot {\left(G_{\varepsilon }\right)}_{i,j}^{k}$$

$$K_{i,j}^{k}=\frac{1}{I^{k}\left(r_{i},r_{j},t\right)}\int_{0}^{t}I^{k}\left(r_{i},r_{j},t^{\prime }\right)\cdot I^{k}\left(r_{i},r_{j},t-t^{\prime }\right)$$

(2c) $$=\frac{\left|r_{i}-r\right|+\left|r-r_{j}\right|}{4\pi D^{k}\left|r_{i}-r\right|\cdot \left|r-r_{j}\right|}\exp\left[\frac{{\left(r_{i}-r\right)}^{2}-{\left(\left|r_{i}-r\right|+\left|r-r_{j}\right|\right)}^{2}}{4D^{k}t}\right]$$

where *I^k^*(*r_i_*, *r_j_*, *t*) is the three-dimensional solution of the diffusion equation to describe the probability of energy transport from *r_i_* to *r_j_* after a time *t* at load step *k* [23]. The parameters *c^k^* and *D^k^* are the direct wave velocity and diffusion constant. The values of *c^k^* are acquired through the measurement of the first arriving *P* and *S* wave signals, and the *D^k^* value is calculated by best fitting the diffusion solution to collected coda waveform. A preliminary study of their calculations is given in [16], and their details are not further discussed here for the sake of brevity.

### 4.3. Inverse Algorithms

At each load step *k,* estimating *σ*(*r*) and *m*(*r*) values from all available (i.e., *N_m_* = 4 × 20) *DC* and *dv*/*v* results defines the inverse problem. To solve it, the concrete beam is discretized into elementary cells (voxels) of size *dV* = 3 × 3 × 3 cm^3^ with the total cell number *N_c_* = 199 × 29 × 10, which implies that the spatial resolution of imaging results is up to a few centimeters. It should be noted that smaller cell will lead higher resolution but increase computation time, and operators can select the size at their own convenience. Each cell centered on the position *r* is considered as a candidate location for the apparition of the changes. Becasue *N_m_* ≪ *N_c_* corresponding to undetermined systems, an iterative, least-square algorithm initially proposed by Tarantola et al. [24] and further developed by Larose et al. as *Locadiff* technique [19,20] is applied

(3a) $${\left(\sigma^{k}\right)}^{n}={\left(\sigma^{k}\right)}^{n-1}+{\left[{\left(G_{DC}^{k}\right)}^{T}\cdot {\left(C_{DC}^{k}\right)}^{-1}\cdot \left(G_{DC}^{k}\right)+{\left(C_{\sigma }^{k}\right)}^{-1}\right]}^{-1}\cdot {\left(G_{DC}^{k}\right)}^{T}\cdot {\left(C_{DC}^{k}\right)}^{-1}\cdot \left[DC^{k}-\left(G_{DC}^{k}\right)\cdot {\left(\sigma^{k}\right)}^{n-1}\right]$$

(3b) $${\left(m^{k}\right)}^{n}={\left(m^{k}\right)}^{n-1}+{\left[{\left(G_{\varepsilon }^{k}\right)}^{T}\cdot {\left(C_{\varepsilon }^{k}\right)}^{-1}\cdot \left(G_{\varepsilon }^{k}\right)+{\left(C_{m}^{k}\right)}^{-1}\right]}^{-1}\cdot {\left(G_{\varepsilon }^{k}\right)}^{T}\cdot {\left(C_{\varepsilon }^{k}\right)}^{-1}\cdot \left[{\left(dv/v\right)}^{k}-\left(G_{\varepsilon }^{k}\right)\cdot {\left(m^{k}\right)}^{n-1}\right]$$

where

(3c) $$C_{DC}^{k}={\left(Noise_{i,j}^{k}\cdot DC_{i,j}^{k}\right)}^{2}\delta \;\mathrm{and}\;C_{\varepsilon }^{k}={\frac{1-\left(1-DC_{i,j}^{k}\right)}{2\left(1-DC_{i,j}^{k}\right)}}^{2}\cdot \frac{6\sqrt{\pi /2}}{f_{\Delta }\cdot \left(2\pi f\right)\cdot t^{3}}\delta$$

(3d) $$C_{\sigma }^{k}=C_{m}^{k}=\left(std^{k}\cdot \frac{\sqrt[{3}]{dv}}{L_{c}{}^{k}}\right)\cdot \exp\left(-\frac{\left|r-r^{\prime }\right|}{L_{c}{}^{k}}\right)$$

Now, *σ*(*r*) and *m*(*r*) are written as column vectors of length *N_c_*. *G^k^_DC_* and *G^k^_ε_* are *N_m_* × *N_c_* matrices, where each row corresponds to sensitivity kernel of a source-receiver pair. The superscripts *T* and *n* indicate the matrix transposition and iteration number.

In Equation (3c), *C^k^_DC_* and *C^k^_ε_* describe the errors on the *DC^k^_ij_* and *(dv*/*v)^k^_ij_* results. The observations from each source-receiver pair are independent, making *C^k^_DC_* and *C^k^_ε_* diagonal matrices of size *N_m_* × *N_m_*. *Noise^k^_i,j_* and *f_∆_* are the relative fluctuations (i.e., noise levels) and frequency bandwidth of wave signals.

In Equation (3d), *C^k^_σ_* and *C^k^_m_* are *N_c_* × *N_c_* covariance matrices following exponential formulations in concrete [25], which define the correlations of *σ*(*r*) and *m*(*r*) estimation values between cells *|r−r’|* and between iterative steps. *L^k^_c_* is the typical correlation distance and *std^k^* is the allowed model fluctuation. These two tunable parameters present a compromise between the simplicity of the estimation and the quality of the fit in the inversion algorithms. Their values are determined through the *L*-curve method [26]; i.e., *std^k^* ≈ 5.6 × 10^−4^λ*^k^* and *L^k^*_c_ ≈ 16*λ^k^*, where *λ^k^* denotes the central wavelength.

Here, the initial (*σ^k^*)^0^ and (*σ^k^*)^0^ models are zero vectors, since there is no any priori information about changes. The calculation time of the inverse procedures with total 200 iterations was about 1.5 h by running Matlab codes on a personal desktop equipped with i7-4770 CPU and 16 GB memory.

## 5. Results

### 5.1. Structural Analysis and Gauge Measurement

**Figure 5:**
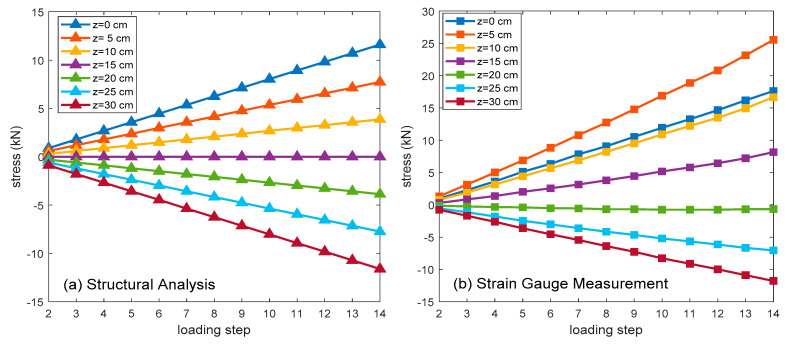
The analysis and measurement stress changing values along the section of L/2.

The stresses that were obtained from gauge measurements and structural analyses are firstly presented to denote the general conditions of the concrete beam. Figure 5a shows the analysis results of a healthy T-beam along the L/2 section at each load step, and the associated gauge measurements are indicated in Figure 5b. As might be expected, the upper (*z* = [20, 25, 30] cm) and lower (*z* = [0, 5, 10] cm) portions encounter compression and tension forces that increase with loads.

Because the lower portions of the beam contain several pre-existing subtle cracks that reduce its stiffness and capacity, the measurement values at *z* = [0, 5, 10] cm, in general, are larger than the analysis values and the non-linear load-stress relationships appear. The measured height of the neutral axis is raised to about z = 20 cm, whereas the analysis result is at z = 15 cm. On the contrary, the gauges that are positioned in the upper portion are far from the cracks. As a result, their measurement and analysis values are close, and the linear load-stress relationship holds.

### 5.2. Imaging Results

**Figure 6:**
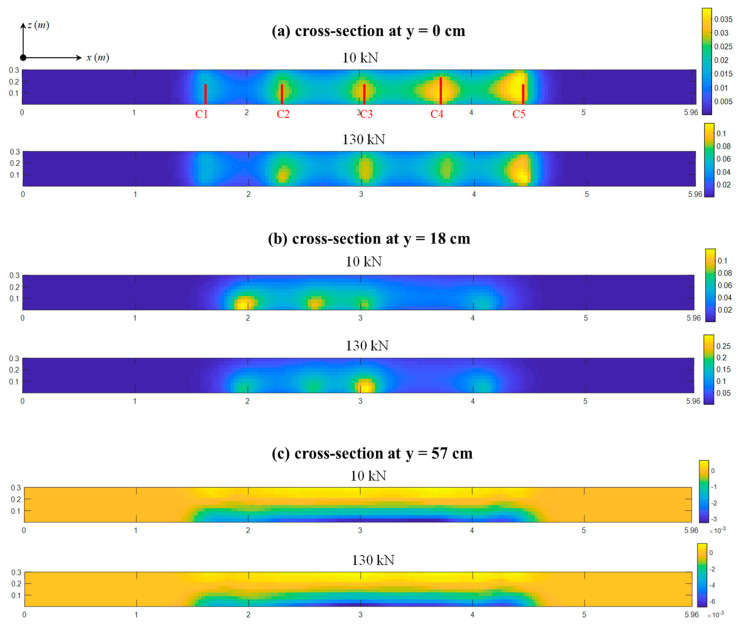
The σ(r)-based imaging results under 10 kN and 130 kN at (**a**) y = 0, (**b**) y = 18, and (**c**) y = 57 cm.

**Figure 7:**
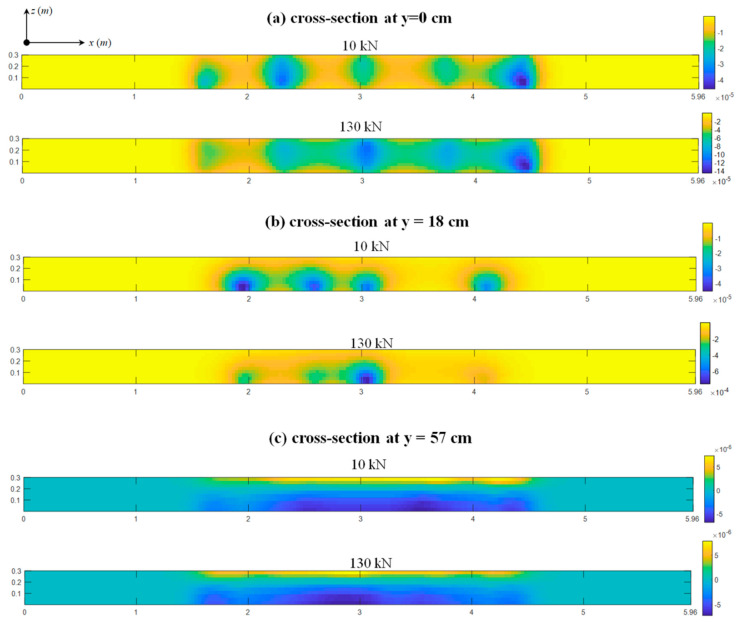
The m(r)-based imaging results under 10 kN and 130 kN at (**a**) y = 0, (**b**) y = 18, and (**c**) y = 57 cm.

For each load step k, the *σ*(*r*) and *m*(*r*) values at each elementary cell were calculated through Equation (3), which results in three-dimensional imaging of local changes in micro-structures and velocities. Figure 6 and Figure 7 display three sets of the *σ*(*r*)- and *m*(*r*)-based images at the cross-sections of *y* = [0, 18, 57] cm, where each set is comprised of two images under the 10 kN (minimum) and 130 kN (maximum) loads.

Large *σ*(*r*) and *m*(*r*) values are spatially concentrated in five regions where the pre-existing cracks C1~C5 exist, as shown in Figure 6a and Figure 7a. The sizes of these regions are partially consistent with the extent of the cracking; e.g., the wider crack C5 induces the larger concentration area around it. Subsequently, at the cross-section of *y* = 18 cm (Figure 6b and Figure 7b), both *σ*(*r*)- and *m*(*r*)-based images show four changing regions but the crack C5 is undetectable. This result is again in accord with the actual measurements that the depth of the crack C5 at the base is only about 9 cm, see Table 1.

Recall that the measured depths of all cracks are smaller than 57 cm. Consequently, there are no obvious concentration (i.e., cracking) effects found in Figure 6c and Figure 7c, and their *σ*(*r*) and *m*(*r*) values decrease significantly when compared to their associated results at y = [0, 18] cm. By subtracting the *σ*(*r*) and *m*(*r*) values at the beam’s neutral axis, the imaging results are consistent with typical stress distributions of a four-bending test expected from classical mechanics. Specifically, the upper and lower portions are in compressive and tensile stresses, as well as both ends remain almost stress-free, and these results also agree well with the structural analysis and gauge measurements in Figure 6.

At all cross-sections, as loads increase from 10 kN to 130 kN, the internal stresses increase and the changes in each local structure also increase due to the slight extension of the pre-existing cracks. Thus, the scales of *σ*(*r*) and *m*(*r*) values generally increase by a factor of 2~3. Note that the absence of sensors on side y = 87 cm also weaken the resolutions of deep cross-section (e.g., y = 57 cm). The study of this phenomenon requires sensor networks placing at both webs of the beam, which might be an issue worth of research in future work.

The results that are shown in Figure 6 and Figure 7 prove that both *DC-* and *dv*/*v*-inversed images can localize subtle cracks with a high degree of accuracy and display the distributions of stress changes at the cross-section where cracking effects are weak. This finding is in accordance with the CWI theory; i.e., damages play a predominant effect on coda waveform variations by introducing extra scatters and more tortuous propagation paths, as well as intensify the non-homogeneous stress distributions in concrete.

## 6. Conclusions

In conclusion, this paper presents an experimental study of three-dimensionally imaging local changes in concrete by the application of inverse algorithms to coda wave measurements. When applied to a large reinforced concrete beam, the joint effects of its pre-existing cracks and external stresses on coda waves have been monitored while using a network of fixed transducers placed at the surface. The global waveform decorrelations and velocity variations are firstly evaluated through coda wave interferometry (CWI) technique. Then two inverse algorithms are independently applied to map the densities of changes at each localized position. The imaging results display the stress changes and subtle cracks for both temporal and spatial domains. The presented study might promote the development of concrete structural nondestructive evaluation and testing (NDT) techniques.

Here, we note that coda wave interferometry is directly applied to full waveforms during whole propagation periods. By selecting appropriate source-receiver pairs and time-windows, it is possible to extract information about stress change distribution even if with existing cracks, which leave an important future work. Furthermore, the inversion images reflect stress changing distributions by mapping decorrelation and velocity densities; i.e., the pixel value corresponds to CWI parameters (*σ*(*r*) and *m*(*r*)) not real stresses. Approaches to produce stress-based images are being studied by introducing acoustoelastic theory on CWI parameter-stress value conversions.

## Figures and Tables

**Table 1:** Crack geometry (unit: mm).

Micro-Cracks	C1	C2	C3	C4	C5
length at y = 0	170	160	170	240	160
width at y = 0	0.12	0.25	0.12	0.15	0.22
depth at z = 0	130	280	470	300	90
